# Hyperphosphataemia and NADPH Oxidase Regulation in Pathophysiological Processes: Implications for Oxidative Stress and Disease Progression

**DOI:** 10.3390/antiox14040461

**Published:** 2025-04-12

**Authors:** Marco Antonio Lacerda-Abreu, José Roberto Meyer-Fernandes

**Affiliations:** Instituto de Bioquímica Médica Leopoldo de Meis, Universidade Federal do Rio de Janeiro, Rio de Janeiro 21941-901, RJ, Brazil

**Keywords:** hyperphosphataemia, NADPH oxidase, oxidative stress

## Abstract

Hyperphosphataemia is a key contributor to oxidative stress (OS) and cellular dysfunction across various pathological conditions. While numerous studies have associated phosphate overload with redox imbalances, the role of NADPH oxidase (NOX) in this process has received limited attention. NOX enzymes are major enzymatic sources of reactive oxygen species (ROS), and their activation has been implicated in the progression of chronic kidney disease, vascular calcification, metabolic disorders, and cancer development. Under hyperphosphataemic conditions, excessive ROS production exacerbates endothelial dysfunction, promotes vascular smooth muscle cell transdifferentiation, induces chronic inflammation, and facilitates tumour progression. Despite increasing evidence linking phosphate metabolism to NOX activation, the underlying molecular mechanisms remain poorly characterised. This review critically examines the relationship between hyperphosphataemia and NADPH oxidase-mediated OS and explores its impact on disease pathophysiology. By providing an integrated analysis of the current findings, this work aims to highlight the pathological consequences of phosphate-induced OS and identify potential therapeutic strategies to mitigate its effects.

## 1. Introduction

Inorganic phosphate (Pi) is a fundamental chemical element that plays a crucial role in ATP synthesis through glycolysis and oxidative energy metabolism. Additionally, it serves as an essential component of DNA, RNA, phospholipids, and various phosphorylated metabolic intermediates [1,2]. The recommended dietary allowance for phosphorus in healthy adults is at least 700 mg per day, although this requirement varies depending on dietary intake [3]. An imbalance in phosphate homeostasis can have severe pathological consequences [4]. Hyperphosphataemia, characterised by elevated serum Pi levels, is recognised as a major contributor to morbidity and mortality in patients with chronic kidney disease (CKD) [5].

Hyperphosphataemia can be classified as mild (1.44–1.76 mmol/L), moderate (1.76–2.08 mmol/L), or severe (>2.08 mmol/L) on the basis of the serum Pi concentration [6]. Moreover, accumulating evidence has linked hyperphosphataemia to hormonal imbalances and the progression of various diseases. This is primarily attributed to phosphate overload disrupting cellular signalling pathways and inducing oxidative stress, ultimately exacerbating disease pathology [3,7].

Oxidative stress (OS) is the alteration of redox homeostasis in favour of reactive oxygen species (ROS) and the depletion of the enzymatic and non-enzymatic antioxidant system [8,9]. ROS, including superoxide radicals (O_2_^•−^), hydroxyl radicals (^•^OH), hydrogen peroxide (H_2_O_2_), and peroxynitrite (ONOO^−^), are key oxidants involved in redox signalling. Among these, H_2_O_2_ and O_2_^•−^ play crucial roles as redox signalling molecules, which are primarily generated by the mitochondrial electron transport chain (ETC), xanthine oxidase, and NADPH oxidases (NOX) [8,9].

NADPH oxidase exhibits rapid responsiveness, primarily through the stimulation of various growth factors, hormones, and signalling pathways [10]. The NOX enzyme family represents a key group of enzymes responsible for ROS production, with seven isoforms expressed in different tissues in most mammals, particularly humans (Table 1) [11]. The physiological and pathological roles of NOX isoforms have been reviewed over the past two decades, including detailed analyses of their structure, regulation, and tissue distribution [12,13]. All NOX family enzymes are transmembrane proteins that traverse the membrane six times and generate O_2_^•−^ from molecular oxygen via NADPH oxidation and a haem-dependent mechanism [14]. Superoxide can subsequently be converted into H_2_O_2_ by superoxide dismutase (SOD), or it may be directly generated by certain NOX isoforms, thereby playing crucial roles in redox signalling and OS regulation [15]. Given its involvement in OS-related pathology, NOX inhibition has been proposed as a potential therapeutic strategy to mitigate oxidative damage in various disease conditions [14,15].

Although the association between hyperphosphataemia and OS has been demonstrated [3], the specific contribution of NADPH oxidase to this process has received limited attention. Understanding this interplay is crucial for elucidating how hyperphosphataemia contributes to disease pathophysiology. This review aims to analyse the available evidence of NADPH oxidase-mediated OS in the context of hyperphosphataemia, highlighting its implications for chronic kidney disease, vascular calcification, metabolic disorders, and cancer. Table 2 compiles experimental models exploring NOX activation under hyperphosphataemic conditions across various diseases, providing a basis for the discussion developed throughout this review.

## 2. Chronic Kidney Disease

Extracellular matrix accumulation in the glomeruli and tubulointerstitial area is well recognised as a major factor in the development of glomerulosclerosis and interstitial fibrosis, both of which are structural markers of renal disease progression [26,27]. Clinical and experimental evidence suggests that hyperphosphataemia is not only a consequence of CKD but may also play a direct role in its development [5]. Even a modest increase in serum phosphate levels has been shown to induce fibrosis in the renal interstitial matrix by stimulating the synthesis of the interstitial matrix protein fibronectin via activation of the ERK1/2 and AKT signalling pathways [28].

5′ Adenosine monophosphate-activated protein kinase (AMPK) is a serine/threonine kinase that serves as a key metabolic sensor in nearly all eukaryotic cells [29]. Its activity and subunit composition vary by cell type, with the α1- and α2-subunits expressed in the kidney and glomerular mesangial cells [30]. In immortalised human mesangial cells (iHMCs), AMPK inhibition by high glucose levels is associated with extracellular matrix (ECM) accumulation. This process is mediated by the activation of NOX4, an isoform of NADPH oxidase, which stimulates transforming growth factor β-1 (TGFβ-1) signalling, thereby promoting ECM deposition [31].

**Figure 1:**
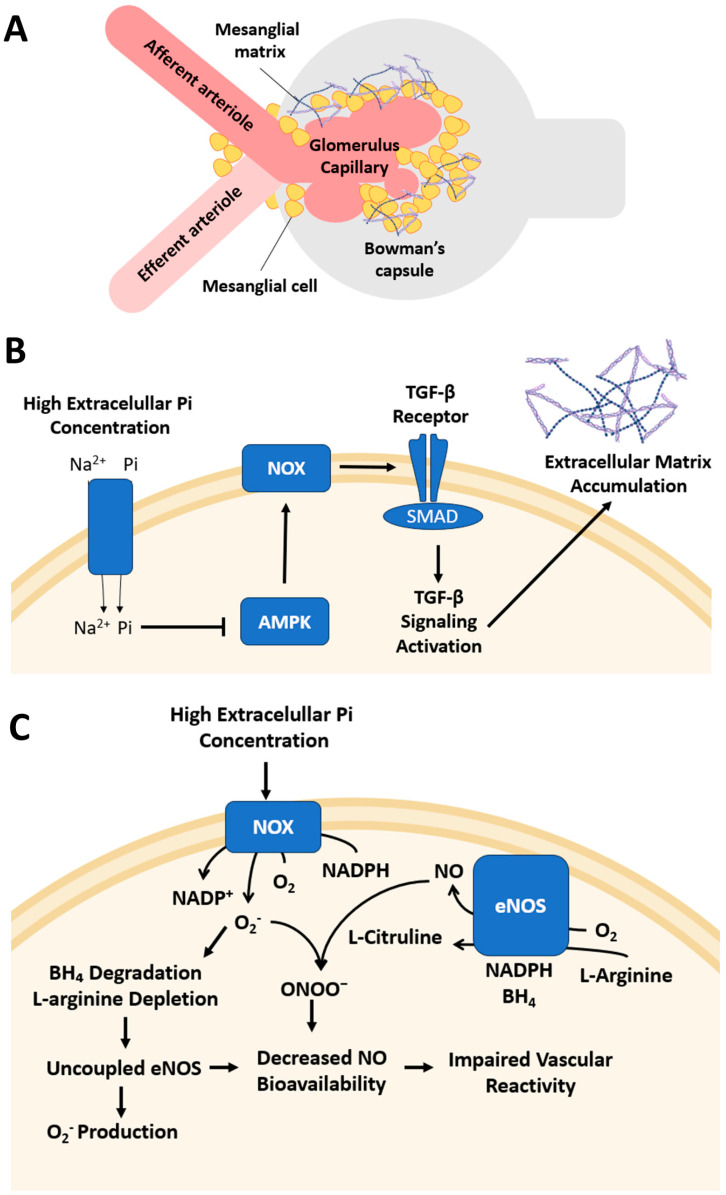
Hyperphosphataemia-induced extracellular matrix accumulation in the glomerulus. (**A**) Schematic representation of the glomerulus, highlighting mesangial cells, glomerular capillaries, and afferent/efferent arterioles. (**B**) In mesangial cells, high extracellular Pi levels induce extracellular matrix accumulation via AMPK inhibition and activate NOX, which stimulates TGF-β/SMAD signalling. Arrows indicate stimulation, and dashes indicate inhibition. (**C**) In endothelial cells of afferent and efferent arteries, high extracellular Pi levels activate NOX, increasing O_2_^−^ production and NADPH consumption. Superoxide reacts with NO to form ONOO^−^, reducing NO bioavailability. In parallel, oxidative stress promotes BH_4_ degradation and L-arginine depletion, leading to eNOS uncoupling and further superoxide generation. The resulting reduction in NO availability impairs endothelial function, contributing to vascular dysfunction in CKD.

In CKD, hyperphosphataemia at a concentration of 5 mM Pi contributes to disease progression by promoting ECM accumulation in mesangial cells (Table 2) [16]. AMPK inactivation by hyperphosphataemia induces OS via NOX4-dependent NADPH oxidase activation, increasing ROS production and triggering TGFβ-1 signalling through SMAD3 phosphorylation, which promotes ECM accumulation in human mesangial cells [16]. Consequently, this cascade leads to glomerulosclerosis and interstitial fibrosis, both of which are hallmark features of CKD progression. These processes are illustrated in Figure 1, which presents the NOX-dependent mechanisms induced by hyperphosphataemia in renal cells. AMPK activation with AICAR prevents these effects by reducing NOX4 expression. Likewise, pharmacological inhibition of NADPH oxidase or NOX4 silencing also alleviates phosphate-induced alterations. These interventions may represent promising strategies to limit the progression of hyperphosphataemia-associated chronic kidney disease [16].

Although hyperphosphataemia promotes ECM accumulation in renal compartments, its effects on vascular tissue appear to follow a distinct pathway. TGF-β signalling is known to induce the expression of matrix metalloproteinases (MMPs), a mechanism demonstrated in CKD models under hyperphosphataemic conditions. In this context, increased phosphate levels promote the expression of MMP-2 and MMP-9 in vascular tissue, contributing to ECM degradation and facilitating the development of vascular calcification [32].

Alterations in the balance between ROS and nitric oxide (NO) are critical in the pathophysiology of CKD. NADPH oxidases are a major source of superoxide in renal tissue, and their activation under hyperphosphataemic and pro-inflammatory conditions leads to increased ROS production [33]. This oxidative environment impairs NO bioavailability via two main mechanisms: (i) chemical inactivation, where superoxide reacts with NO to form ONOO^−^; and (ii) uncoupling of endothelial nitric oxide synthase (eNOS), triggered by oxidative degradation of the cofactor tetrahydrobiopterin (BH_4_) and depletion of L-arginine, thereby shifting the enzyme from NO production to superoxide generation [33]. Moreover, hyperphosphataemia has been shown to directly downregulate eNOS expression and activity, further compromising NO-mediated vascular regulation [34]. These combined mechanisms impair vascular reactivity in afferent and efferent arterioles, contributing to glomerular hypertension and the progression of CKD [33,34].

## 3. Vascular Calcification

In addition to its role in CKD progression, hyperphosphataemia is also implicated in systemic complications, most notably vascular calcification (VC), a pathological process frequently observed in patients with impaired renal function. VC is characterised by the deposition of phosphate calcium crystals in blood vessels, the myocardium, and cardiac valves and is closely associated with genetic disorders, diabetes, CKD, and ageing. This progressive condition significantly contributes to increased morbidity and mortality worldwide [35].

Studies investigating the response of aortic smooth muscle cells to elevated phosphate levels (>1.4 mM Pi), which are comparable to those observed in CKD patients, have demonstrated a dose-dependent increase in calcium deposition in cell cultures [35]. This phosphate-induced mineralisation is accompanied by the upregulation of osteogenic markers, including osteocalcin and core-binding factor-1 (Cbfa1/Runx2), the latter of which is recognised as a key transcription factor essential for osteoblast differentiation [36]. Furthermore, OS and the resulting endothelial dysfunction have been identified as major contributors to the pathogenesis of atherosclerosis and cardiovascular disease (CVD) [37,38]. Notably, NADPH oxidase, particularly the NOX4 isoform, has been implicated as a significant source of ROS in vascular dysfunction [39].

**Figure 2:**
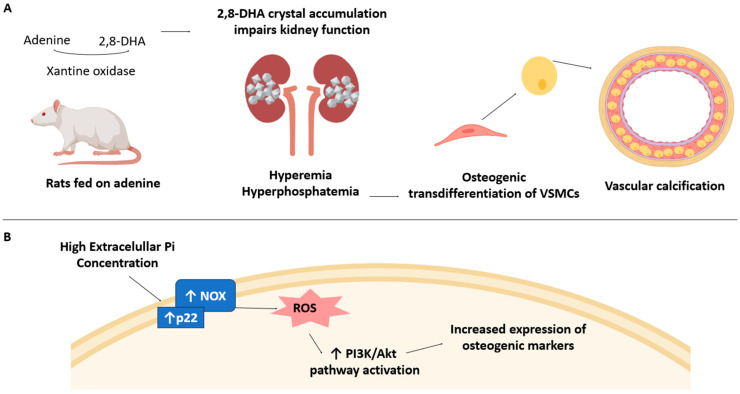
Hyperphosphataemia-induced vascular calcification and NOX-mediated osteogenic differentiation. (**A**) Rats fed on adenine develop kidney dysfunction due to the accumulation of 2,8-dihydroxyadenine (2,8-DHA) crystals, leading to hyperphosphataemia and hyperaemia. Elevated phosphate levels promote the transdifferentiation of VSMCs into an osteogenic phenotype, ultimately contributing to vascular calcification. (**B**) Increased Pi levels upregulate NOX and its subunit p22, leading to elevated ROS production. This oxidative stress activates the PI3K/Akt signalling pathway, which promotes the expression of osteogenic markers, driving vascular calcification. The arrows indicate stimulation.

In a rat model fed an adenine-rich diet, elevated serum phosphorus levels were observed, which were positively correlated with the calcium content in the aorta, indicating vascular calcification [40]. As shown in Figure 2, Panel A, the adenine-induced kidney injury model demonstrates that hyperphosphataemia, a consequence of impaired renal function, is strongly associated with VC, as increased serum phosphate levels promote the osteogenic transdifferentiation of vascular smooth muscle cells (VSMCs) into osteoblast-like cells [40]. In this context, studies on uraemic rats have demonstrated that elevated extracellular phosphate levels drive vascular calcification by upregulating NOX4 and its p22 subunit, leading to increased ROS production (Table 2) [17,18]. This phosphate-induced OS facilitates osteogenic transdifferentiation, as evidenced by the upregulation of osteogenic markers, including CBFA1, OSX, E11, DMP-1, and SOST, alongside the downregulation of the VSMC markers α-SMA and SM22α. Figure 2, Panel B, illustrates how the PI3K/AKT signalling pathway plays a central role in this process, as H_2_O_2_ stimulates the tyrosine phosphorylation of PI3K, leading to AKT activation—a key step in osteoblast-like transdifferentiation [17,18].

Furthermore, Boehme et al. [18] demonstrated that antioxidants such as TEMPOL (a membrane-permeable nitroxide radical scavenger) and TIRON (a cell-permeable superoxide scavenger) can attenuate pro-calcific effects under hyperphosphataemic conditions, as observed in CKD, emphasising their potential therapeutic relevance in the prevention of phosphate-induced VC [18].

Given the central role of NOX4 in phosphate-induced OS and vascular calcification, strategies to counteract its effects have been explored. In primary human aortic smooth muscle cells (HAoSMCs), fibulin-3, an extracellular matrix glycoprotein, has been implicated in the inhibition of vascular OS and remodelling in hypertension (Table 2) [19]. Notably, phosphate treatment significantly upregulated NOX4 and CYBA, key components of the NADPH oxidase system involved in superoxide generation and OS. However, fibulin-3 was shown to suppress the phosphate-induced expression of the osteogenic transcription factors MSX2 and CBFA1, as well as the chondrogenic transcription factor SOX9. Additionally, it reduced the expression and enzymatic activity of alkaline phosphatase (ALP) in VSMCs, thereby mitigating vascular calcification [19]. These findings suggest that fibulin-3 plays a protective role against phosphate-induced vascular calcification by counteracting NOX4-mediated OS and osteogenic transdifferentiation.

### 3.1. Dextromethorphan as an NADPH Oxidase Inhibitor

One recent study examined the role of dextromethorphan (DXM) in hyperphosphataemia-induced VC in Wistar rats with adenine-induced renal failure and diet-induced hyperphosphataemia [20]. DXM, which is recognised primarily as an N-methyl-D-aspartate (NMDA) receptor agonist, is the dextrorotatory isomer of the codeine analogue levorphanol, a morphinan [41]. However, DXM has also been identified as an NADPH oxidase antagonist [42]. In VSMCs, hyperphosphataemia leads to a reduction in ATP levels and the mitochondrial membrane potential, effects that are attenuated by DXM treatment (Table 2) [20]. By inhibiting NADPH oxidase, DXM reduces ROS production and mitigates phosphate-induced VSMC calcification, further underscoring the role of NADPH oxidase in OS and vascular calcification under hyperphosphataemic conditions [20].

### 3.2. Role of Macrophages Activated in Vascular Calcification

Age-related vascular calcification is a major independent predictor of morbidity and mortality in CVD patients [43]. A key contributor to this process is intimal calcification, a hallmark of atherosclerosis that is closely associated with macrophage infiltration in lipid-rich regions of atherosclerotic plaques [44]. As atherosclerosis progresses, distinct macrophage subtypes emerge at different disease stages, influencing the calcification process [45]. Macrophages contribute to intimal calcification by secreting factors that promote the osteogenic differentiation and mineralisation of VSCMs. However, this effect is phenotype-dependent, as alternatively activated macrophages (M2φs) exhibit anti-calcifying properties, whereas classically activated macrophages (M1φs) are associated with pro-calcifying activity [46].

Elevated phosphate concentrations stimulate the activation of nonpolarised macrophages (M0φs) into a distinct phenotype resembling alternatively activated macrophages (M2φs), termed phosphate-activated macrophages (MPiφs) [21]. This phenotypic shift is characterised by a 3.4-fold downregulation of *NOX1*, the gene encoding NADPH oxidase 1, a key enzyme involved in ROS production in macrophages (Table 2) [21]. Concurrently, MPiφs exhibit a marked upregulation of antioxidant enzymes, including superoxide dismutase 3 (9.3-fold), peroxiredoxin 1 (3.8-fold), glutathione peroxidase 1 (1.6-fold), catalase (1.4-fold), and superoxide dismutase 2 (1.3-fold). These findings suggest that hyperphosphataemia induces an antioxidant response in macrophages characterised by reduced NADPH oxidase activity and increased antioxidant enzyme expression, likely as a protective mechanism against OS [21].

## 4. Ageing-Related Vascular Dysfunction

Vascular function is maintained through the coordinated secretion of molecules that regulate endothelial homeostasis via autocrine and paracrine signalling. Among these, NO plays a central role in preserving vascular integrity, alongside redox balance and endothelin-1 (ET-1) [47]. However, ageing induces structural and functional alterations in the arterial wall, disrupting vascular cell behaviour and increasing susceptibility to dysfunction [48]. In this context, OS and inflammation are key pathological processes, as evidenced by multiple studies in both healthy older adults and rodent models, highlighting their contributions to endothelial impairment and vascular ageing [49,50].

Hyperphosphataemia has been shown to impair endothelial function. In adenine-induced kidney disease models, dietary phosphate restriction not only reduces hyperphosphataemia but also improves aortic vasodilation, suggesting that lowering phosphate levels can restore vascular function [51].

Furthermore, a study comparing young (5-month-old) and aged (24-month-old) C57BL/6 mice fed a diet containing 0.6% phosphate or 0.2% phosphate demonstrated that age-associated hyperphosphataemia promotes vascular dysfunction by impairing endothelium-dependent relaxation and increasing inflammation and vascular fibrosis (Table 2) [22]. In aged mice, hyperphosphataemia was correlated with a significant increase in ROS production and elevated Nox4 expression in the aorta compared with young mice. Additionally, aged mice exhibited reduced expression of Nrf2 and the antioxidant enzymes Sod2-Mn and Gpx1, indicating that hyperphosphataemia contributes to age-related vascular dysfunction by activating Nox4, thereby promoting redox imbalance and OS [22]. Antioxidants, such as N-acetylcysteine (NAC), have been shown to effectively suppress ROS production and reduce pathological processes such as inflammation and vascular fibrosis, supporting their potential therapeutic use in conditions characterised by NOX-mediated OS induced by hyperphosphataemia [22].

## 5. Renal Osteodystrophy

Renal osteodystrophy (ROD) is a bone disorder specifically associated with CKD and is characterised by alterations in bone turnover, mineralisation, volume, linear growth, and strength due to disturbances in calcium, phosphate, parathyroid hormone (PTH), and vitamin D metabolism [22]. The term renal osteodystrophy was first introduced in 1943, nearly 60 years after the initial observation of a link between kidney disease and skeletal abnormalities [52]. Historically, ROD encompassed all bone manifestations in patients with CKD or end-stage kidney disease (CKD G5D). However, it is now recognised as part of the broader chronic kidney disease–mineral and bone disorder (CKD-MBD) spectrum, which includes systemic mineral imbalances, vascular calcification, and soft tissue complications in addition to skeletal abnormalities [52].

Hyperphosphataemia plays a pivotal role in CKD-MBD [53]. Excessive Pi retention in the body can impair bone mineralisation, dysregulate cellular signalling, and promote cell death [3,53]. These disturbances contribute to systemic mineral and bone metabolism dysfunction, leading to skeletal growth defects, increased bone fragility, and pathological calcification, particularly vascular calcification [53].

In osteoblastic MC3T3-E1 cells, phosphate enhances ROS production, a process mediated by NADPH oxidase, as demonstrated by the inhibitory effects of apocynin and diphenyleneiodonium (DPI) (Table 2) [23]. Silencing of the NADPH oxidase isoforms Nox1 and Nox4 significantly reduces phosphate-induced ROS production, confirming their role as key mediators in this process [23].

Furthermore, phosphate disrupts osteoblast differentiation by reducing the alkaline phosphatase (ALP) activity induced by bone morphogenetic protein 2 (BMP2) and downregulating the expression of osteoblastic marker genes, including ALP, osteocalcin, and runt-related transcription factor 2 (Runx2) [23]. This inhibitory effect is reversed by NADPH oxidase inhibition, suggesting that phosphate-induced OS contributes to the osteodystrophy observed in hyperphosphataemic conditions, such as CKD [23].

## 6. Atrial Fibrillation

Atrial fibrillation (AF) was first described over a century ago, and its clinical significance, along with that of its related arrhythmia, atrial flutter, has continued to grow due to the increasing incidence of this disease and its associated morbidity and mortality, including cerebrovascular accidents [54]. AF is the most commonly diagnosed arrhythmia and is characterised by rapid, continuous electrical activity in the atria, leading to impaired atrial contraction and reduced efficiency in blood delivery to the ventricles. This results in rapid ventricular contractions, which contribute to heart failure symptoms and blood stasis in the atria [55].

Electrolyte imbalances have been identified as contributing factors to AF risk. Notably, an increased incidence of AF has been observed in individuals with elevated serum phosphate levels [56]. In 8-week-old male C57BL/6 mice subjected to a high-phosphate diet (2%), elevated phosphate levels increased Nox4 expression and activity, leading to increased ROS production (Table 2) [24]. The resulting OS induced mitochondrial dysfunction, contributing to atrial remodelling and fibrosis [24].

To elucidate the molecular mechanisms underlying this process, phosphoproteomic analysis was performed on human atrial fibroblasts treated with normal (1 mM) or a high (2 mM) Pi concentration for 24 hours (Table 2) [24]. Among the hyperphosphorylated proteins induced by the high Pi concentration, 19 were associated with the NF-κB pathway, and STAT3 was also hyperphosphorylated, highlighting the involvement of two pathways that are known mediators of atrial fibrosis in AF [57,58]. Activation of the NF-κB pathway and STAT3 upregulates Nox4 expression, which in turn promotes collagen synthesis, exacerbating fibrotic remodelling. Furthermore, RNA interference (siRNA)-mediated inhibition of NF-κB, STAT3, and Nox4 significantly reduced collagen expression in response to high phosphate levels, confirming the role of these pathways in Nox4 activation and subsequent fibrosis. These findings suggest that Nox4 is a key mediator of hyperphosphataemia-induced atrial remodelling and increased susceptibility to AF [24].

## 7. Cancer

Cancer cells are inherently self-sufficient in terms of growth-promoting signals; however, accumulating evidence suggests that exogenous nutritional factors, including Pi, play a significant role in tumour progression [59]. As a fundamental nutrient, Pi is an essential component of phospholipids and nucleotides (RNA and DNA) and is crucial for energy metabolism, both as ATP and as a substrate for metabolic intermediates [1]. According to the growth rate hypothesis (GRH), tumour cells exhibit an increased demand for Pi due to their accelerated proliferation, requiring an abundant supply to sustain nucleic acid synthesis and bioenergetic processes [60]. Consistently, studies have reported significantly elevated serum Pi concentrations in cancer patients (2.52 ± 0.72 mmol/L) compared with healthy individuals (1.09 ± 0.19 mmol/L) [61], reinforcing the notion that phosphate metabolism is closely linked to tumour growth.

Dysregulation of Pi homeostasis in cancer cells has been implicated in metabolic reprogramming, favouring oncogenic signalling pathways that promote proliferation, invasion, and survival [1]. High dietary phosphate intake has also been associated with tumorigenesis in various cancers, including lung and skin malignancies, where Pi has been shown to activate oncogenic pathways such as the Akt and N-Ras pathways, thereby enhancing cellular transformation and tumour development [62,63].

Lacerda-Abreu et al. [25] demonstrated that H_2_O_2_ generation is a cellular response to elevated Pi concentrations in breast cancer cells. In their study, 1 mM Pi was used as a control, representing serum Pi levels in healthy individuals, whereas 2 mM and 4 mM Pi corresponded to concentrations observed in cancer patients. The most pronounced regulatory effects were observed at 8 mM Pi. Notably, in triple-negative breast cancer (TNBC) cells (MDA-MB-231), higher Pi concentrations (2, 4, and 8 mM) induced H_2_O_2_ production, an effect not observed in other breast cell lines, such as MCF-10A and MCF-7 (Table 2) [25,64].

It is well established that more than 50% of a cell’s endogenous ROS are generated by the mitochondrial electron transport chain (ETC) and transmembrane NOX [65]. In MDA-MB-231 cells, Pi-induced H_2_O_2_ production is mediated by two distinct sources, depending on the exposure duration [25]:

(1) Short-term exposure (1 h): Pi hyperpolarises the mitochondrial membrane, increases mitochondrial ROS production, inhibits O_2_ consumption, and enhances PKC activity [25].
(2) Long-term exposure (24 h): The source of Pi-induced H_2_O_2_ production shifts from mitochondria to NADPH oxidase. Using the NOX inhibitor VAS2870, Lacerda-Abreu et al. [63] demonstrated that this compound effectively inhibited H_2_O_2_ production only during prolonged Pi exposure but not in the short term, confirming the role of NOX as the primary ROS source under sustained Pi elevation [25].

However, the precise mechanism by which NADPH oxidase is activated under hyperphosphataemic conditions remains to be clarified. The authors reported that short-term exposure to elevated Pi levels induced H_2_O_2_ production, which was associated with the activation of PKC [25]. Notably, PKC contains two pairs of zinc-finger motifs within its regulatory domain, which are susceptible to oxidative modification. H_2_O_2_ and other oxidants can disrupt the structural integrity of these zinc fingers, relieving autoinhibition and resulting in a catalytically active PKC, independent of Ca^2+^ or phospholipids [66]. To further investigate this mechanism, PKC activation was assessed using phorbol 12-myristate 13-acetate (PMA), a known PKC stimulator, which led to increased H_2_O_2_ production under control conditions. Interestingly, prolonged exposure to hyperphosphataemia induced H_2_O_2_ production to levels comparable to those observed in PMA-treated control cells. Moreover, treatment with VAS2870 effectively suppressed H_2_O_2_ generation induced by both Pi and PMA. These findings suggest that chronic exposure to high Pi levels activates NADPH oxidase via a PKC-dependent mechanism. Furthermore, in response to OS exacerbated by NOX, PKC specifically modulates NFK-B activation to initiate the transcription of genes related to epithelial–mesenchymal transition (vimentin and E-cadherin), a migratory phenotype [25].

## 8. Conclusions

**Figure 3:**
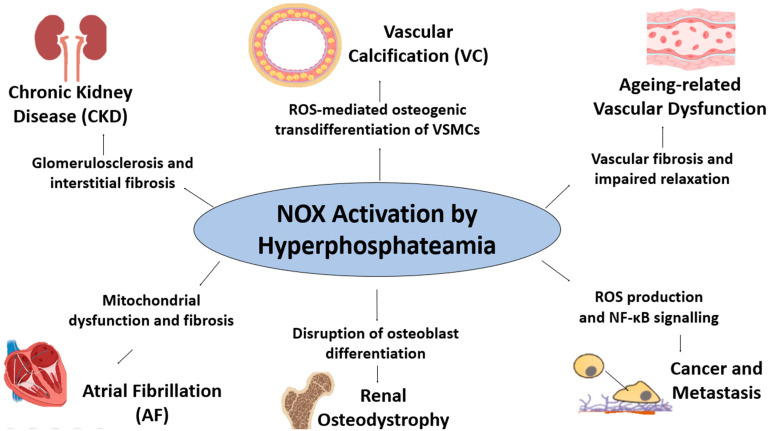
Hyperphosphatemia-induced NOX activation and its pathological consequences. NOX activation increases ROS production, mitochondrial dysfunction, and inflammation, contributing to vascular calcification via osteogenic transdifferentiation of VSMCs, ageing-related vascular dysfunction, glomerulosclerosis and fibrosis in CKD, mitochondrial dysfunction in atrial fibrillation, impaired osteoblast differentiation in renal osteodystrophy, and NF-κB-driven cancer progression and metastasis. The arrows indicate stimulation.

Hyperphosphataemia is increasingly recognised as a key driver of OS across various pathological conditions, with increasing evidence implicating NOX in mediating phosphate-induced redox imbalances. NOX activation under hyperphosphataemic conditions promotes excessive ROS production, exacerbating endothelial dysfunction, VC, chronic inflammation, tumour progression, and renal fibrosis, as summarised in Figure 3. However, further studies are needed to elucidate the signalling pathways underlying NOX activation in response to hyperphosphataemia. By synthesising the current findings, this review lays the groundwork for future research aimed at developing strategies to mitigate phosphate-induced oxidative damage and its pathological consequences.

## Figures and Tables

**Table 1:** NOX isoforms in different cell types and tissues [11].

Isoforms	Subunits	Regulators	Expression Sites
NOX1	p22phox, NOXA1, NOXO1, RAC1	ANG II, PDGF	Colon epithelial cells, vascular smooth cells, endothelial cells, uterus, placenta, osteoclasts, retinal pericytes, and macrophages.
NOX2	gp91phox, p22phox, p40phox, p47phox, p67phox, RAC1	PKC, TNF-α, phosphatidic acid	Phagocytes, vascular cells, endothelium, fibroblasts, cardiomyocytes, skeletal muscle, hepatocytes, and haematopoietic stem cells.
NOX3	p22phox, NOXO1, NOXA1, RAC1	Unknown	Inner ear, lung endothelial cells, foetal spleen, kidney, lung, and skull.
NOX4	P22phox	Poldip2	Kidney, smooth muscle cells, endothelial cells, fibroblasts, keratinocytes, osteoclasts, neurons, and hepatocytes.
NOX5	none	Ca^2+^, ptdlns(4,5)p2	Lymphoid tissues, testes, spleen and endothelial cells.
DUOX1	DUOXA1, DUOXA2	IL-4, IL-3, cAMP, PKA	Thyroid gland, airway epithelia, placenta, prostate, testis, pancreas, and heart.
DUOX2	DUOXA1, DUOXA2	IFN-γ, PLC, PKC	Thyroid gland, airway epithelia, epithelial cells in salivary excretory ducts and rectal glands.

Abbreviations: ANG II—angiotensin II; PDGF—platelet-derived growth factor; PKC—protein kinase C; TNF-α—tumour necrosis factor alpha; Ca^2+^—calcium ion; ptdIns(4,5)P_2_—phosphatidylinositol 4,5-bisphosphate; IL—interleukin; PLC—phospholipase C; PKA—protein kinase A; Poldip2—polymerase delta-interacting protein 2; cAMP—cyclic adenosine monophosphate; IFN-γ—interferon gamma.

**Table 2:** The relationship between hyperphosphataemia and NOX activation—an overview of the disease model.

Disease	Model Used	Pi Concentration	Pi Exposure	Correlation with NOX	Ref.
Chronic Kidney Disease	Immortalised human mesangial cells (iHMCs)	5 mM	24 h	High Pi level induced OS by NOX4 activation following AMPK inhibition.	[16]
Vascular calcification	Male Sprague Dawley (SD) rats	1.2% (diet)	6 weeks	NOX4 was time-dependently upregulated in the aortic media of uraemic rats.	[17]
Vascular calcification	Rat model of adenine-induced CKD and cultured VSMCs	3 mM	14 days	High Pi level increased expression of NOX4 and p22phox, enhancing ROS generation.	[18]
Vascular calcification	Primary human aortic smooth muscle cells (HAoSMCs)	2 mM β-glycerophosphate	24 h	Pi treatment upregulated NOX4 and CYBA, key components of NADPH oxidase.	[19]
Vascular calcification	Wistar rat model with adenine-induced CKD and HASMCs	2.5 mM Pi	14 days	Hyperphosphataemia induced ROS via NOX, leading to vascular calcification.	[20]
Vascular calcification	Mouse bone marrow-derived macrophages (BMDMs)	2.5 mM	7 days	High Pi level led to downregulation of NOX1 in macrophages.	[21]
Vascular dysfunction related to ageing	C57BL6 mice (young: 5 months, old: 24 months).	0.6% (diet)	3 months	Hyperphosphataemia increased NOX4 expression and ROS production.	[22]
Osteodystrophy	Osteoblastic murine MC3T3-E1 cells	5 mM	42 h	Pi increased ROS production through NOX1 and NOX4.	[23]
Atrial Fibrillation	8-week-old male C57BL/6 mice	2% (diet)	10 weeks	Elevated Pi level increased NOX4 expression and ROS production.	[24]
Breast cancer	MDA-MB-231 cells.	8 mM	24 h	Pi induced ROS production through PKC-mediated NOX activation.	[25]

## Data Availability

The data that support the findings of this study are available in this review.

## Abbreviations:

AF	Atrial fibrillation
ALP	Alkaline phosphatase
AMPK	5′ Adenosine monophosphate-activated protein kinase
ANG II	Angiotensin II
ATP	Adenosine triphosphate
BMDMs	Bone marrow-derived macrophages
BMP2	Bone morphogenetic protein 2
CBFA1	Core-binding factor alpha 1 (Runx2)
CVD	Cardiovascular disease
CKD	Chronic kidney disease
CKD-MBD	Chronic kidney disease–mineral and bone disorder
cAMP	Cyclic Adenosine Monophosphate
DHA	Dihydroxyadenine
DUOX	Dual oxidase
DUOXA	Dual oxidase activator
DXM	Dextromethorphan
ECM	Extracellular matrix
ET-1	Endothelin-1
ETC	Electron transport chain
Gpx1	Glutathione peroxidase 1
HAoSMCs	Human aortic smooth muscle cells
IFN-γ	Interferon gamma
iHMCs	Immortalised human mesangial cells
IL	Interleukin
M0φs	Nonpolarised macrophages
M1φs	Classically activated macrophages
M2φs	Alternatively activated macrophages
MPiφs	Phosphate-activated macrophages
mRNA	Messenger ribonucleic acid
MSX2	Msh homeobox 2
NADPH	Nicotinamide adenine dinucleotide phosphate
NF-κB	Nuclear factor kappa B
NO	Nitric oxide
NOX	NADPH oxidase
NOXA1	NADPH oxidase activator 1
NOXO1	NADPH oxidase organiser 1
O_2_^•−^	Superoxide anion
OS	Oxidative Stress
^•^OH	Hydroxyl radical
OSX	Osterix (SP7 transcription factor)
Pi	Inorganic phosphate
PKA	Protein kinase A
PKC	Protein kinase C
PLC	Phospholipase C
PMA	Phorbol 12-myristate 13-acetate
Poldip2	Polymerase (DNA-directed), delta interacting protein 2
PTDlns(4,5)P2	Phosphatidylinositol 4,5-bisphosphate
PTH	Parathyroid hormone
RAC1	Ras-related C3 botulinum toxin substrate 1
ROD	Renal osteodystrophy
ROS	Reactive oxygen species
RUNX2	Runt-related transcription factor 2
SMA	Smooth muscle actin
SM22α	Smooth muscle 22 alpha
SMAD	Mothers against decapentaplegic homolog
SOD	Superoxide dismutase
SOX9	SRY-box transcription factor 9
STAT3	Signal transducer activator of transcription 3
TGF-β	Transforming growth factor beta
TNBC	Triple-negative breast cancer
TNF-α	Tumour necrosis factor alpha
VC	Vascular calcification
VSMCs	Vascular smooth muscle cells
